# Mediating role of social support between sleep quality, anxiety and depressive symptoms in Chinese women undergoing in vitro fertilization treatment

**DOI:** 10.1177/2050312120930163

**Published:** 2020-06-10

**Authors:** Ying Cui, Danian Li, Borong Zhou, Yanshan Lin, Yingchun Zeng

**Affiliations:** 1 Department of Psychiatry, The Third Affiliated Hospital of Guangzhou Medical University, Guangzhou, China; 2 Department of Brain Disease, The First Affiliated Hospital of Guangzhou University of Chinese Medicine, Guangzhou, China; 3 Reproductive Medicine Center, The Third Affiliated Hospital of Guangzhou Medical University, Guangzhou, China; 4 Research Institute of Gynecology and Obstetrics, The Third Affiliated Hospital of Guangzhou Medical University, Guangzhou, China

**Keywords:** Social support, anxiety, depressive symptoms, in vitro fertilization treatment, Chinese women, path analysis

## Abstract

**Introduction:**

Infertility is a significant health problem, and the prevalence of infertility among women is increasing in developing countries. This study aims to explore whether social support plays a mediating role in the links between exogenous variables, sleep quality, anxiety, and depressive symptoms in Chinese women undergoing in vitro fertilization.

**Methods:**

This is a cross-sectional study comprising a sample of Chinese women undergoing in vitro fertilization treatment at a tertiary reproductive medicine center located in South China.

**Results:**

The final testing model showed good fit, with normed χ^2^ = 39.317, p = 0.055, comparative fit index = 0.948, Tucker–Lewis index = 0.902, incremental fit index = 0.951, normed fit index = 0.906, root mean square error of approximation = 0.046). The final path model supported the proposed model: partner relationship, a woman’s age, financial strain, duration of infertility, and cycles of in vitro fertilization were exogenous variables for depressive symptoms, while social support was a significant mediator between sleep quality, anxiety, and depressive symptoms.

**Conclusion:**

The empirical support from this study could facilitate the development of appropriate interventions to reduce depressive symptoms, and to promote the mental health of Chinese women undergoing in vitro fertilization treatment.

## Introduction

Infertility is a significant health problem, and the prevalence of infertility is increasing in developing countries such as China.^1,2^ The World Health Organization estimates that 8%–10% of married women experience difficulties in becoming pregnant.^2^ While many women who experience infertility can become pregnant through assisted reproductive technologies (ARTs) such as in vitro fertilization (IVF), comprising more than 99% of ARTs,^3,4^ IVF treatment can result in various psychological-emotional consequences, including stress, anxiety, depression, and hopelessness.^2,5^

IVF is a medical procedure in which a woman’s egg is fertilized with sperm, resulting in an embryo that is then transferred to the woman’s uterus. The hope is that this will be an option for having a biological child.^6^ In preparation for starting and completing an IVF cycle, a woman must dedicate extensive financial and physical resources in hopes of becoming pregnant. Women may experience anxiety and uncertainty, with concerns about whether the IVF treatment will work.^4^ Advanced reproductive age, longer duration of infertility, more than three cycles of IVF treatment, and an unsatisfactory marital relationship could lead to increased levels of anxiety and depression, which could in turn result in lower rates of pregnancy in women undergoing IVF treatment.^3,7^ In addition, recent research examining the relationship between sleep quality and symptoms of depression and anxiety in women has found poor sleep quality to be significantly associated with the highest levels of anxiety and depressive symptoms throughout pregnancy.^8,9^ However, more social support, especially from loved ones, can reduce stress and anxiety levels in women undergoing IVF treatment.^4^

**Figure 1.**
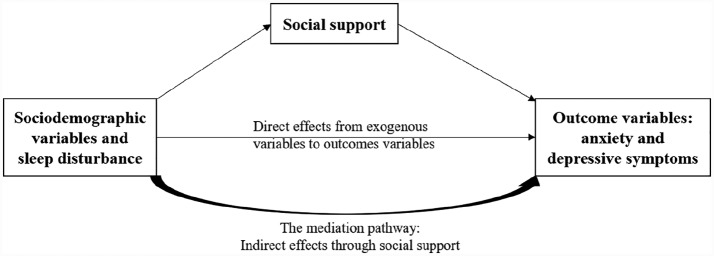
Proposed conceptal model of this study.

Consequently, infertile women share common experiences, including anxiety and depression.^10^ Patel and Sharma Kumar^11^ indicated there are personal, situational, and treatment-related risk factors for infertility distress in terms of anxiety and depressive symptoms. Well-established research has suggested that anxiety and depressive symptoms in infertile women are associated with older age, marital relationship,^12^ sleep disturbance,^8^ and treatment-related factors, such as duration of infertility, medical side effects and failures related to IVF treatment.^13^ Perceived sufficient social support decreases infertile women’s negative emotional symptoms, including anxiety and depression.^14^ In other words, social support may be a protective factor against infertility distress.^15^ Therefore, a proposed conceptual model for this study is that clinical and sociodemographic characteristics are potential exogenous variables; social support may be taken as a potential mediator; and poor sleep disturbance, anxiety, and depressive symptoms represent the psychosocial consequences of undergoing IVF treatment. The proposed conceptual model is depicted in Figure 1.

This study’s aims were to explore whether social support plays a mediating role in the links between sociodemographic and clinical factors, sleep quality, anxiety, and depressive symptoms in Chinese women undergoing IVF treatment. Study hypotheses were (1) sociodemographic and clinical factors would be positively associated with anxiety and depressive symptoms; and (2) social support could mediate the pathways between sleep problems and psychological health, including anxiety and depressive symptoms.

## Materials and methods

### Ethical approval

Ethical approval was obtained from the ethics review committee of The Third Affiliated Hospital of Guangzhou Medical University (ethics approval no. ELKS2020DXH). A research nurse recruited participants one day before embryo transfer (ET) treatment. All women participated on a voluntary basis and gave written informed consent before data collection.

### Study design, sample, and setting

A cross-sectional study of Chinese women with infertility problems undergoing ETs was conducted at a general hospital in Guangzhou, China. The study comprised 458 participants recruited during the previous 12 months. Eligibility criteria for participants were being an adult (18 years or older), undergoing IVF, and in the final IVF phase of ET. Women suffering from a mental disorder, such as psychosis or schizophrenia; women who were not using ART; or who were experiencing a high-risk pregnancy due to maternal (e.g. cardiovascular disease) health issues were excluded. Sample size estimation followed the general principle of “the minimum sample size for studies using structural equitation modeling or path analysis should not be below 200” suggested by Hoe.^16^

### Study measures

A demographic information sheet was used to collect participant sociodemographic characteristics, including age, education level, spousal relationship quality, financial concerns, duration of infertility, and number of IVF cycles.

Social support was measured by the Social Support Rating Scale (SSRS). The SSRS consists of 10 items, with higher scores indicating better social support.^17^ The SSRS is widely used to assess social support for Chinese women.^18,19^ In this study, the internal consistency of SSRS by Cronbach’s alpha was 0.91.

The Chinese version of the Pittsburgh Sleep Quality Index (PSQI) was used to assess participants’ sleep disturbance. The PSQI consists of a 19-item questionnaire used to assess subjective sleep quality, latency and duration; habitual sleep efficiency; sleep disturbances; use of sleeping medication, and daytime dysfunction.^20^ The total PSQI score was calculated by summing the domain scores (ranging from 0 to 21). A PSQI score of ⩽5 is associated with good sleep quality, while a score of >5 is associated with poor sleep quality.^8^ In this study, the internal consistency by Cronbach’s alpha of PSQI was 0.85.

Anxiety symptoms were measured using the Self-rating Anxiety Scale (SAS). The SAS is a 20-item rating scale with a theoretical score range extending from 20 to 80, with scores from 20 to 44 indicating no anxiety; and scores ⩾45 describing anxiety cases.^21^ The SAS Chinese version is widely used in the case of Chinese women during pregnancy.^19^ In this study, the internal consistency by Cronbach’s alpha of SAS was 0.91. Depressive symptoms were measured using the Self-rating Depression Scale (SDS). The SDS is a 20-item rating scale with a theoretical score range extending from 20 to 80, with scores ranging from 20 to 44 indicating no depression, and ⩾45 describing depressive cases.^21^ The SDS Chinese version is widely used in the case of Chinese women during pregnancy.^18,19^ In this study, the internal consistency by Cronbach’s alpha of SDS was 0.93.

### Data collection and statistical analysis

This study was conducted at the outpatient clinic of a South China general hospital’s Reproductive Medicine Center. A total of 500 women was approached, with 458 women joining this study and completing the questionnaire. The data collection period started at the beginning of May 2018 and stopped at the end of September 2018. Data were analyzed using the Statistical Package for Social Sciences (SPSS) for Windows, version 22.0 and R-3.5.1. The descriptive statistics of the findings were summarized by SPSS. A path analytic approach used R to portray the hypothesized causal paths between exogenous variables, sleep quality, anxiety, and depressive symptoms. The overall path model fit was assessed by the following goodness-of-fit indices: a non-significant chi-square value (p > 0.05), normed fit index (NFI ⩾ 0.90), incremental fit index (IFI ⩾ 0.90), Tucker–Lewis index (TLI ⩾ 0.90), comparative fit index (CFI ⩾ 0.90), and root mean square error of approximation (RMSEA ⩽ 0.08).^22^

## Results

**Figure 2.**
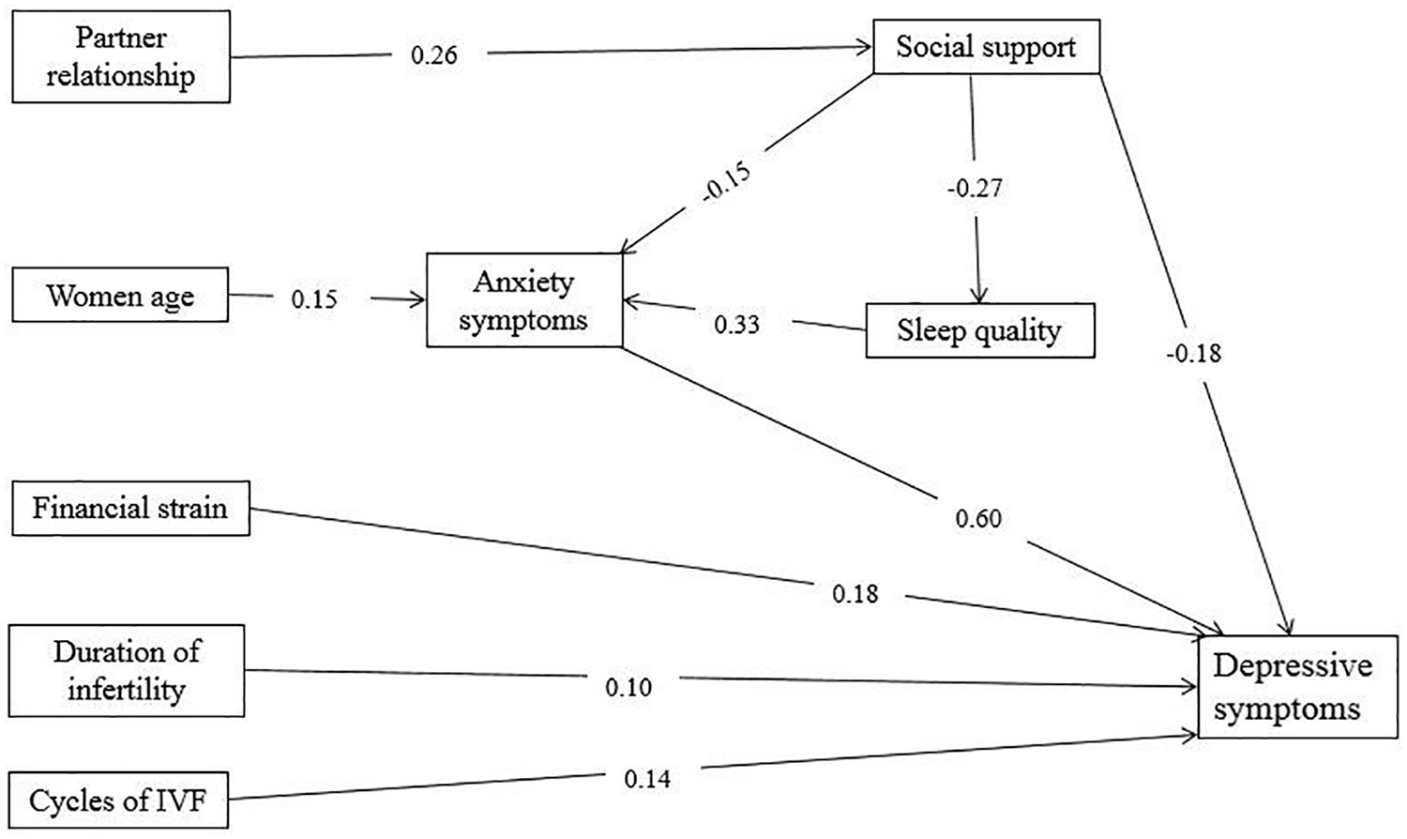
Final path model of this study.

Table 1 presents research participants’ sociodemographic and clinical characteristics, and mean scores of sleep quality, anxiety, and depressive symptoms and social support levels. Nearly one-third of research participants could be described as suffering from possible depression (n = 137, 22.9%). Table 2 summarizes the estimates of standardized direct, indirect, and total effects of significant exogenous variables, sleep quality, anxiety symptoms, and social support on depressive symptoms. Partner relationship quality (0.26) and age (0.15) have indirect effects on depressive symptoms. Mediators of total social support (–0.18) have direct effects on depressive symptoms. Anxiety symptoms (0.60) have a high direct effect on depressive symptoms. A final path model was established, as shown in Figure 2. Standard beta weights were used to represent path coefficients, as presented on each arrow in Figure 2. All path coefficients are significant at the level of p < 0.05. This path model yielded a good fit for exogenous variables, mediators of sleep quality, anxiety and depressive symptoms in Chinese women undergoing IVF treatment (χ^2^ = 39.317, p = 0.055, CFI = 0.948, TLI = 0.902, IFI = 0.951, NFI = 0.906, RMSEA = 0.046).

**Table 1.** Demographic and clinical features of participants (N = 458).

Variables	Mean (SD)	n (%)
Age (y)	32.07 (4.98)	
Duration of infertility (y)	3.75 (2.49)	
No. of IVF cycles	1.66 (0.61)	
Education levels (y)	12.93 (3.41)	
Partner relationship		
Good		405 (88.4)
General		53 (11.6)
Financial strain		
Yes		105 (22.9)
No		353 (77.1)
Sleep quality (total PSQI score)	6.07 (2.72)	
Good (⩽5)		80 (46.1)
Poor (>5)		378 (53.9)
Anxiety (total SAS score)	41.64 (9.25)	
No anxiety (<45)		160 (34.9)
Anxiety (⩾45)		298 (65.1)
Depression (total SDS score)	46.81 (10.65)	
No depression (<45)		226 (49.3)
Depressive symptoms (⩾45)		507 (50.7)
Social support (total SSRS score)	37.90 (7.49)	

IVF: in vitro fertilization; PSQI: Pittsburg Sleep Quality Index; SAS: Self-rating Anxiety Scale; SDS: Self-rating Depression Scale; SSRS: Social Support Rating Scale.

**Table 2.** Summary of the direct, indirect, and total effects of significant factors on depressive symptoms among Chinese women (N = 458).

Variables	Effects	Partner relationship	Significant factors						
			Women age	Financial strain	Duration of infertility	Cycles of IVF	Sleep quality	Anxiety symptoms	Social support
Depressive symptoms	Direct	0.00	0.00	0.18	0.10	0.14	0.33	0.60	−0.18
	Indirect	0.26	0.15	0.00	0.00	0.00	0.00	0.000	0.000
	Total	0.26	0.15	0.18	0.10	0.14	0.33	0.60	−0.18

IVF: in vitro fertilization.

## Discussion

A conceptual model of sociodemographic variables, mediators of social support, and depressive symptoms has been established for Chinese women undergoing IVF treatment. The final path model supported this proposed model with goodness-of-fit indices. Partner relationship quality, a woman’s age, financial strain, duration of infertility, and IVF cycles were exogenous variables for depressive symptoms, while social support was a significant mediator between sleep disturbance and anxiety and depressive symptoms.

In accordance with previous research,^19^ social support has direct mediating effects on exogenous factors, sleep quality, anxiety, and depressive symptoms. Other research has also confirmed that women who receive social support during pregnancy or when preparing for pregnancy could be protected from stressful events.^23^ This study’s findings are also in line with psychosocial stress theories, which state that social support is a positive mediator for depressive and anxiety symptoms.^24,25^ In addition, the indirect effects on depressive symptoms of a woman’s age, partner relationship quality and sleep quality indicate that advanced reproductive age, a poor partner relationship and poor sleep quality are possible sociodemographic causative factors. Better management of these causative factors could reduce depressive symptoms in women undergoing IVF treatment.

This study’s findings have implications for reproductive care practice, with the ultimate goal of reducing depressive symptoms in women undergoing ARTs treatment, including IVF. Longer duration of infertility, repeated IVF failures and financial strain all have a direct effect on increased depressive symptoms. Hence, relevant interventions should be developed to help women cope more effectively with these stressors. As greater social support for women undergoing IVF treatment can mediate the relationship between sleep quality, anxiety and depressive symptoms, promoting social support is a possible strategy to enhance reproductive care services.

Study strengths include the use of widely validated and reliable instruments—such as social support, sleep quality, and anxiety and depressive symptoms—to measure study variables, as previously seen in multiple studies of Chinese women.^18,19,26^ In addition, this study used multivariate analysis to test the links between exogenous variables, mediating variables, and outcome variables of depressive symptoms by taking potential confounders into account. This study’s limitation was the cross-sectional nature of the data; causal inference or directionality can be precluded, despite the final path model with “cause” and “effect” variables. Further research—for example, conducting a prospective and longitudinal study to examine the relationship between study variables in a predictive and robust way—is required. Another limitation of this study is lacking of precise sample size calculation.

While this study has several limitations, its findings have the following implications. This study adopted a path analysis assessing the direct and indirect effects of sociodemographic, clinical, sleep quality and social support factors on infertility distress in women undergoing IVF treatment. Among infertile women, financial stress and treatment-related factors, such as IVF cycles, were significantly related to depressive symptoms. This study suggests that early identification of women at risk would enable the provision of timely social support, to help women manage potential emotional problems and meet their reproductive goals.^27–29^ It confirms that fertility problems are typically accompanied by significant emotional distress and that healthcare providers should focus psychosocial resources on infertile women who are in greatest need of support.^14,27^ Finally, emotional problems could negatively affect IVF outcomes. Therefore, future research should explore the potential relationship between infertility distress and IVF outcomes.^27^

## Conclusion

This study examined a pertinent conceptual model of sleep quality, anxiety, mediators of social support, and depressive symptoms. This path model has enriched theories on the determinants of depressive symptoms in Chinese women undergoing IVF treatment. The empirical evidence found in this study that could facilitate the development of appropriate interventions to reduce depressive symptoms and promote the mental health of Chinese women undergoing IVF treatment through increasing social support, especially from their partners.

## Supplemental Material

430Supplementary_file_of_data_collection_tools_2 – Supplemental material for Mediating role of social support between sleep quality, anxiety and depressive symptoms in Chinese women undergoing in vitro fertilization treatment Click here for additional data file. Supplemental material, 430Supplementary_file_of_data_collection_tools_2 for Mediating role of social support between sleep quality, anxiety and depressive symptoms in Chinese women undergoing in vitro fertilization treatment by Ying Cui, Danian Li, Borong Zhou, Yanshan Lin and Yingchun Zeng in SAGE Open Medicine
