# Supplementary material for: Mediating role of social support between sleep quality, anxiety and depressive symptoms in Chinese women undergoing in vitro fertilization treatment
Source: SAGE Open Med. 2020 Jun 10;8:2050312120930163. doi: 10.1177/2050312120930163 (PMC7288797; doi:10.1177/2050312120930163)
Supplement: 430Supplementary_file_of_data_collection_tools_2 – Supplemental material for Mediating role of social support between sleep quality, anxiety and depressive symptoms in Chinese women undergoing in vitro fertilization treatment [file 430Supplementary_file_of_data_collection_tools_2.pdf]

English translation of the questionnaires
Part 1: Chinese version of Social Support Rating Scale (SSRS)

Guidance: The following questions are used to reflect the support you get in the society. Please write according to the specific requirements of each question and according to your actual situation. Thanks for your cooperation.

1. How many close friends do you have support and help for? (Choose only one)

A No one B 1-2 C 3-5 D 6 or more

2. In the past year, you: (choose only one)

(1) Stay away from family members and live alone.

(2) The residence often changes, and most of the time live with strangers.

(3) Live with classmates, colleagues or friends.

(4) Live with family.

3. You and your neighbor: (choose only one)

(1) Never care about each other, just nodding.

(2) If you encounter difficulties, you may be a little concerned.

(3) Some neighbors care about you.

(4) Most neighbors care about you.

4. You and your colleagues: (choose only one)

(1) Never care about each other, just nodding.

(2) If you encounter difficulties, you may be a little concerned.

(3) Some colleagues care about you.

(4) Most colleagues care about you.

5. Support and care from family members (choose the appropriate option among the four options of none, very little, general, full support)

I. Couple (lover)

A no, B very few, C average, D fully support

II. Parents

A no, B very few, C average, D fully support

III. Children

A no, B very few, C average, D fully support

IV. Siblings

A no, B very few, C average, D fully support

V. Other members (such as sister-in-law)

A no, B very few, C average, D fully support

6. In the past, when you encountered an emergency, the sources of financial support and help in solving practical problems that you once received were:

(1) No source.

(2) The following sources: (multiple choices)

A. Spouse; B. Other family members; C. Relatives; D. Friends; E. Colleagues; F. Work units; G. Official or semi-official organizations such as party and trade unions; H. Unofficial organizations such as religious and social groups; Other (please list)

7. In the past, when you encountered emergencies, the sources of comfort and concern you have received are:

(1) No source.

(2) The following sources (multiple choices)

A. Spouse; B. Other family members; C. Friends D. Relatives; E. Colleagues; F. Work units; G. Party or trade unions and other official or semi-official organizations; H. Unofficial organizations such as religious and social groups; (Please list)

8. The way you talk when you are in trouble: (choose only one)

(1) Never tell anyone.

(2) Only 1-2 people who are very close to each other are involved.

(3) If a friend asks, you will say it.

(4) Actively tell your own troubles to get support and understanding.

9. How to ask for help when you are in trouble: (choose only one)

(1) Just rely on yourself and do not accept help from others.

(2) Rarely ask others for help.

(3) Sometimes ask others for help.

(4) When there are difficulties, often ask for help from family, friends and organizations.

10. For groups (such as party organizations, religious organizations, trade unions, student unions, etc.), you:

(1) Never participate

(2) Occasional participation

(3) Regular participation

(4) Actively participate in and actively participate in activities.

Part 2: Chinese version of the Pittsburgh Sleep Quality Index (PSQI)

Guidance: The following questions are about your sleep in the past month, please select or fill in the answer that best matches your actual situation in the past month. please answer the following question!

１.In the past month, usually go to bed at night ____ o'clock.

２. In the past month, it usually takes ____ minutes from going to bed to fall asleep.

３. nearly 1 month, usually get up in the morning ____

４. In the past 1 month, usually sleep ____ hours per night (not equal to bed time).

For the following questions, please choose the one that suits you best.

５.In the past 1 month, I was troubled by the following conditions affecting sleep:

a. Difficulty falling asleep (can't fall asleep within 30 minutes) (1)None (2) <1 time / week (3)1-2 times / week (4)≥3 times / week

b. Easy to wake up or wake up early at night (1)None (2) <1 time / week (3)1-2 times / week(4)≥3 times / week

c. Go to the toilet at night (1)None (2) <1 time / week (3)1-2 times / week(4)≥3 times / week

d. Breathlessness (1)None (2) <1 time / week (3)1-2 times / week(4)≥3 times / week

e. High cough or snoring sound (1)No (2) <1 time / week (3)1-2 times / week(4)≥3 times / week

f. Feeling cold (1)None(2) <1 time / week (3)1-2 times / week(4)≥3 times / week

g. Feeling hot (1)None(2) <1 time / week (3)1-2 times / week(4)≥3 times / week

h. Nightmare (1)None(2) <1 time / week (3)1-2 times / week(4)≥3 times / week

i. Pain and discomfort (1)None(2) <1 time / week (3)1-2 times / week(4)≥3 times / week

j. Other things that affect sleep (1)None(2) <1 time / week 1-2 times / week(4)≥3 times / week

If yes, please specify:

6.In the past 1 month, in general, you think your sleep quality is very good (2) better (3) poor (4) very poor

7.In the past 1 month, you have been hypnotized with drugs (1)None (2) <1 time / week 1-2 times / week (4)≥3 times / week

8.Do you often feel sleepy in the last month? (1)None(2) <1 time / week (3)1-2 times / week (4)≥3 times / week

9.In the past month, have you lacked the energy to do things (1)No (2)Occasionally there are (3) Sometimes (4)Often there are

Part 3: Self-rating Anxiety Scale (SAS)

1. I feel more nervous or anxious than usual
2. I'm scared for no reason
3. I am easily upset or frightened
4. I think I might be going crazy
5. I think everything is fine
6. My hands and feet are shaking
7. I am troubled by headache, neck pain and back pain

8. I feel vulnerable and tired
9. I feel calm and easy to sit quietly
10. I feel my heart beating fast
11. I am distressed by the dizziness
12. I have a fainting episode, or feel faint
13. I feel easy to inhale and exhale
14. My hands and feet are numb and tingling
15. I am troubled by stomachache and indigestion
16. I often have to urinate
17. My hands and feet are often dry and warm
18. I blush and get hot
19. I fall asleep easily and sleep well all night
20. I have nightmares

Part 4: Self-rating Depression Scale (SDS)

1. I feel depressed and depressed
2. I feel the best mood in the morning
3. I want to cry or want to cry
4. I don't sleep well at night
5. I eat as much as usual
6. My sexual function is normal
7. I feel weight loss
8. I am troubled by constipation
9. My heart beats faster than usual
10. I feel tired for no reason
11. My mind is as clear as usual
12. I don't feel difficult to do things as usual
13. I am restless and unable to keep calm
14. I feel hopeful for the future
15. I'm more irritated than usual
16. I find it easy to decide
17. I feel that I am a useful and indispensable person
18. My life is meaningful
19. If I die, others will live better
20. I still love what I usually like
